# Second harmonic generation in monolithic gallium phosphide metasurfaces

**DOI:** 10.1515/nanoph-2024-0177

**Published:** 2024-07-11

**Authors:** Muyi Yang, Maximilian A. Weissflog, Zlata Fedorova, Angela I. Barreda, Stefan Börner, Falk Eilenberger, Thomas Pertsch, Isabelle Staude

**Affiliations:** Institute of Solid State Physics, 539151 Friedrich Schiller University Jena, Max-Wien-Platz 1, 07743 Jena, Germany; Institute of Applied Physics, Abbe Center of Photonics, 539151 Friedrich Schiller University Jena, Albert-Einstein-Str. 15, 07745 Jena, Germany; Max Planck School of Photonics, Hans-Knöll-Straße 1, 07745 Jena, Germany; Group of Displays and Photonics Applications, Carlos III University of Madrid, Avda. de la Universidad, 30, Leganés, 28911 Madrid, Spain; Fraunhofer-Institute for Applied Optics and Precision Engineering IOF, Albert-Einstein-Straße 7, 07745 Jena, Germany

**Keywords:** gallium phosphide, nonlinear metasurfaces, second harmonic generation, nanofabrication

## Abstract

Gallium phosphide (GaP) offers unique opportunities for nonlinear and quantum nanophotonics due to its wide optical transparency range, high second-order nonlinear susceptibility, and the possibility to tailor the nonlinear response by a suitable choice of crystal orientation. However, the availability of single crystalline thin films of GaP on low index substrates, as typically required for nonlinear dielectric metasurfaces, is limited. Here we designed and experimentally realized monolithic GaP metasurfaces for enhanced and tailored second harmonic generation (SHG). We fabricated the metasurfaces from bulk (110) GaP wafers using electron-beam lithography and an optimized inductively coupled plasma etching process without a hard mask. SHG measurements showed a high NIR-to-visible conversion efficiency reaching up to 10^−5^, at the same level as typical values for thin-film-based metasurface designs based on III–V semiconductors. Furthermore, using nonlinear back-focal plane imaging, we showed that a significant fraction of the second harmonic was emitted into the zeroth diffraction order along the optical axis. Our results demonstrate that monolithic GaP metasurfaces are a simple and broadly accessible alternative to corresponding thin film designs for many applications in nonlinear nanophotonics.

## 1 Introduction

III–V semiconductors play an important role in nonlinear nanophotonics due to their excellent linear and nonlinear optical properties [1], [2], [3], [4]. Their relatively high refractive index at optical frequencies allows strong confinement of light and support of Mie-type resonances in structures of sub-wavelength scale [5], [6], [7], [8]. Moreover, their characteristic non-centrosymmetric zincblende crystal structure leads to a high second order nonlinear susceptibility (*χ* ^(2)^) [9], thus supporting the full range of second-order nonlinear processes. While the non-birefringent refractive index poses a challenge for phase matching in the bulk [10], the subwavelength thickness of typical resonant nanostructures alleviates this issue. As such photonic nanostructures supporting efficient second-harmonic generation (SHG) [11], sum-frequency generation [12], [13], and spontaneous parametric down-conversion [14], [15], [16], [17] have been demonstrated. Among the zincblende type III–V semiconductors, gallium phosphide (GaP) has unique advantages due to its large indirect band gap (2.24 eV), which makes the material non-absorptive for light with a wavelength larger than 550 nm [18], thus providing a wide transparency window for efficient generation of visible second harmonic (SH) light [19]. Together with a *χ* ^(2)^ value exceeding 70 pm/V and a refractive index above 3 in the near-infrared spectral range, GaP features an exceptionally favorable combination of material properties for nonlinear nanophotonics. This has already been exploited for efficient wavelength conversion in various nanoscale structures that provide a resonant field enhancement to increase nonlinear-optical responses, such as nanocylinders [18], [20], [21], [22], waveguides [23], [24], [25], photonic crystals [26], nanopillars [27], [28], and metasurfaces [29], [30].

Among the investigated structures, metasurfaces stand out by their ability to tailor the wavefront and polarization of the generated SH wave by spatial variability of the metasurface design [2]. However, common nonlinear metasurface designs rely on single crystalline thin films of the semiconductor material on low-refractive-index substrates to ensure the desired confinement of the optical modes in the final fabricated nanostructures. For GaP, such thin films have been mostly obtained using metal-organic chemical vapor deposition in combination with transfer bonding [22], [23], [29], [31], [32]. While this method results in high-quality single-crystalline films, it is extremely expensive. Alternatively, GaP films can be grown on silicon or sapphire; however, the crystal quality suffers from the lattice mismatch in particular as the film thickness exceeds tens of nanometers [33], [34]. As another, potentially scalable fabrication technique for thin single-crystalline GaP films, ion-beam irradiation in combination with field-assisted thermal bonding has been demonstrated recently [35]. However, this method also requires large-scale expensive equipment. The resulting poor availability of high-quality GaP thin films is severely limiting the investigation of metasurfaces and other photonic nanostructures based on GaP.

Apart from this central issue, two additional complications associated with the design and fabrication of GaP nanostructures need to be considered. Firstly, common schemes for GaP nanofabrication require the use of a metallic hard mask for dry etching, which complicates the fabrication process [18], [25]. Secondly, the specific symmetry of the nonlinear susceptibility tensor of zincblende-type crystals renders SH emission into the normal direction challenging for nanostructures made from (100)-oriented wafers [3], [36]. As shown for GaAs and AlGaAs nanostructures, this can be overcome by choosing a different crystal orientation instead [13], [37], [38].

Here, we propose and demonstrate metasurfaces made from a bulk GaP wafer with (110) crystal orientation, that can overcome all the issues mentioned above. The cylindrical nanoresonators forming the metasurfaces are designed such that they support well-confined Mie-type resonances in the near-infrared spectral range despite the presence of the high-refractive-index GaP substrate. We experimentally fabricate these metasurfaces using a relatively simple procedure combining electron-beam lithography (EBL) and dry etching. The resulting metasurfaces show competitive conversion efficiency and significant SH emission into the normal direction.

## 2 Design and numerical analysis

**Figure 1:**
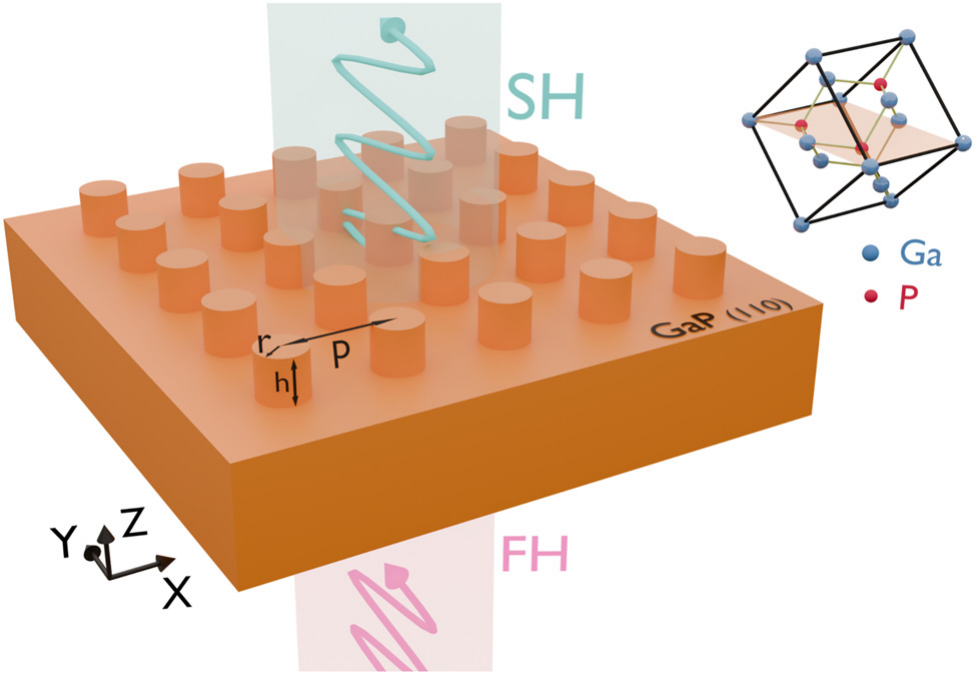
Schematic diagram of the monolithic GaP metasurface, when the fundamental harmonic is incident from the substrate side and the SH is collected in the forward direction. The left (right) inset illustrates the metasurface and the GaP crystal orientation, respectively.

In order to optimize monolithic GaP metasurfaces for SHG, we performed numerical simulations using the finite element method as implemented in the RF module of the commercial software COMSOL Multiphysics 5.6 (see Section 6 for details) [39]. The investigated metasurface consists of a square lattice of cylindrical nanoresonators on bulk GaP substrate having (110) crystal orientation as shown in Figure 1. The crystal axis [001] is parallel to the substrate surface and also parallel to one of the lattice directions of the metasurface. Here we assume it to be along the *x*-axis of the Cartesian coordinate system. The geometry parameters were carefully optimized for maximizing the local enhancement of the fundamental harmonic’s (FH’s) electric field inside the resonator, as detailed below. As a result, the ratio between the radius (*r*), height (*h*), and lattice period (*p*) of the nanocylinders is chosen to be 1:3.6:6.4 to reach a high field enhancement at resonance. The resonant wavelength can be tuned by adjusting the geometric dimensions while keeping the ratio fixed.

**Figure 2:**
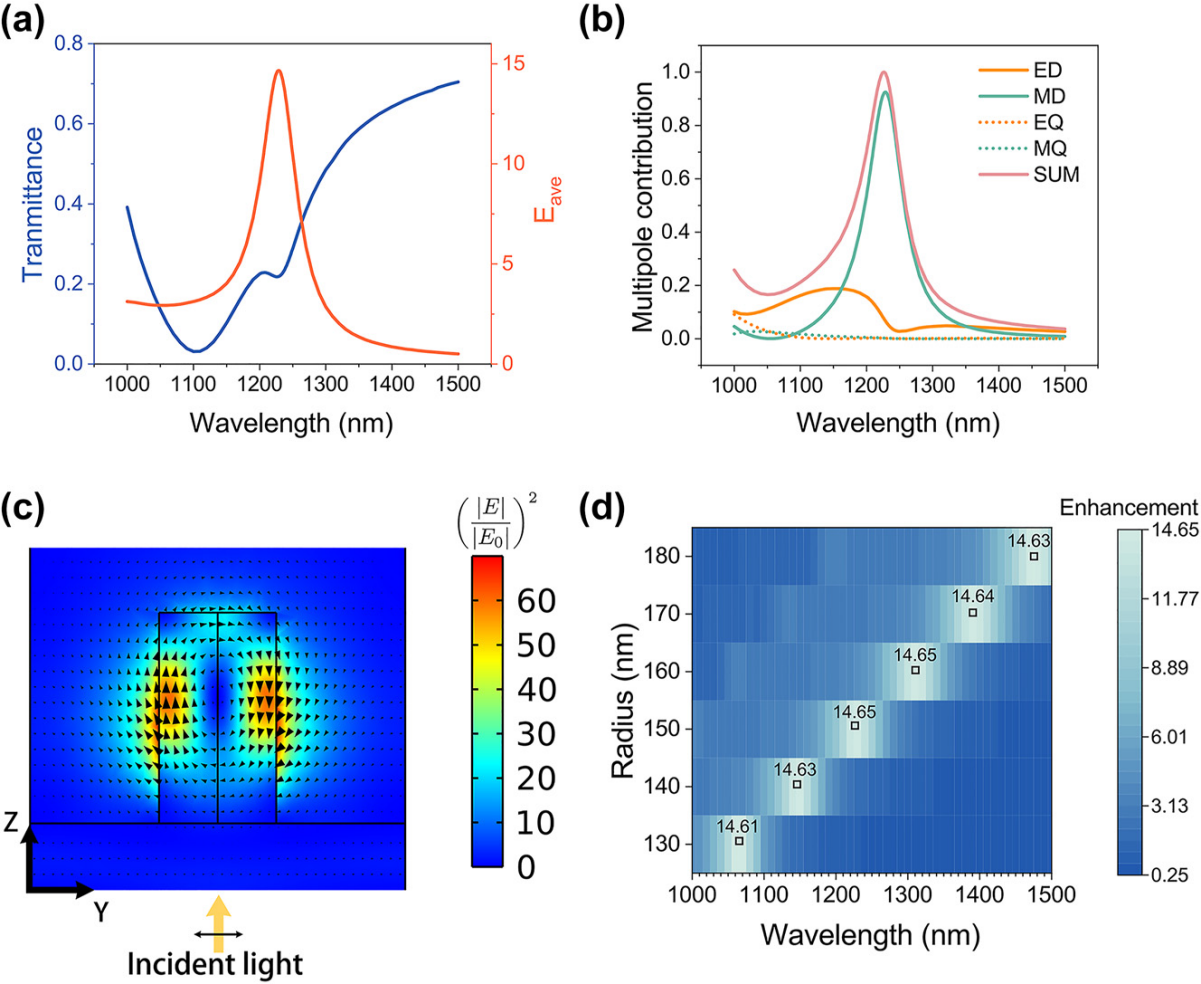
Linear simulation results of the geometrically optimized monolithic GaP metasurface. (a) Transmission spectrum (blue curve) and average electric field enhancement *E*_ave_ inside the nanocylinder (red curve) for a GaP metasurface on a GaP substrate, with a unit cell composed of nanocylinders with *r* = 150 nm and *h* = 540 nm. The period is *p* = 960 nm. (b) Normalized multipole decomposition of the linear electric field inside the nanoresonator. ED stands for electric dipole, MD for magnetic dipole, MQ for magnetic quadrupole, EQ for electric quadrupole, and SUM for the sum of all the multipoles. (c) A cross-section of the electric field enhancement ${\left(\frac{\left|\vec{E}\right|}{\left|\vec{E_{0}}\right|}\right)}^{2}$ in the *yz* plane through the centre of a nanocylinder of the metasurface at a wavelength of 1,230 nm, the peak of the enhancement curve in (a). The arrows size and direction denote the value of ${\left(\frac{\left|\vec{E}\right|}{\left|\vec{E_{0}}\right|}\right)}^{2}$ and direction of the electric field vector, respectively. The outline of the nanocylinder is indicated by a black solid line. The incident light direction and polarization are shown below. (d) *E*_ave_ spectrum of constant ratio of the fixed geometric parameters (*r*:*h*:*p* = 1:3.6:6.4). The boxes and values near the maximum of enhancement spectra show the peak position and enhancement value for each radius.

To generate SH at visible wavelengths longer than the absorption edge of the GaP material, we targeted metasurface designs supporting FH resonances in the near-infrared spectral range around a wavelenth of or in excess of 1100 nm. Figure 2a shows the numerical linear transmission spectrum for a GaP metasurface on a GaP substrate, with a unit cell composed of a nanocylinder with a radius of 150 nm. It is illuminated by a plane wave propagating from the substrate side and linearly polarized along the *y*-axis (blue curve). Note that illumination from the back is more effective in enhancing the electric field in the resonator than from the front (see Supplementary Material for a comparison of front and back illumination). The nanocylinder height and lattice period are 540 nm and 960 nm, respectively, to keep the optimized ratio. The transmission exhibits a broad minimum centered at the wavelength of 1,100 nm. Another smaller minimum appears around 1,230 nm. To quantify the enhancement of the electric field associated with the observed resonances, we further calculated the wavelength-dependent average electric field enhancement *E*_ave_, and defined as [37]

$$E_{\text{ave}}=\frac{1}{V}∭{\left(\frac{\left|\vec{E}\right|}{\left|\vec{E_{0}}\right|}\right)}^{2}\text{d}V$$

where $\vec{E}$ is the amplitude of the electric field and $\vec{E_{0}}$ is the corresponding electric field amplitude in the air corresponding to the incident intensity ( $\vec{E_{0}}=\sqrt{\frac{2I_{0}}{c\epsilon_{0}}}$ ; *I*_0_ is the incident light intensity, *c* is the speed of light and *ϵ*_0_ is the vacuum permittivity). The integration is performed over the entire volume of the nanocylinder [40]. These results are shown in Figure 2a (red curve). The enhancement exhibits a single maximum around 1,230 nm, which corresponds to the position of the small minimum in the transmittance. No significant enhancement is observed at the position of the broad minimum. To understand the nature of the excited resonances, we performed a multipolar analysis of the electric field induced in the nanocylinders. The results are shown in Figure 2b. A pronounced magnetic dipole (MD) resonance exists at a wavelength of 1,230 nm, corresponding to the maximum of the E-field intensity enhancement. We furthermore calculated the mode profile associated with this resonance. The corresponding cross-section image of the normalized electric field, ${\left(\frac{\left|\vec{E}\right|}{\left|\vec{E_{0}}\right|}\right)}^{2}$ , in the *yz*-plane through the centre of the nanocylinder shows a circulating electric field, consistent with a MD resonance. For wavelengths shorter than 1,250 nm, we also recognize a significant contribution of the electric dipole (ED), which becomes dominant for wavelengths below 1,150 nm. This allows us to identify the broad minimum in the transmittance spectrum as an ED resonance. Notably, despite the presence of the high-refractive-index GaP substrate, the optimized metasurface geometry supports the excitation of localized Mie-type resonances in connection with a significant average electric-field intensity enhancement reaching 14.64. While this is in accordance with previous research about monolithic lithium niobate metasurfaces (LiNbO_3_) [37], the higher refractive index of GaP as compared to LiNbO_3_ facilitates 40 % stronger enhancement.

In Figure 2d, we changed the radius while keeping the ratio of the geometric parameters constant (*r*:*h*:*p* = 1:3.6:6.4) and simulated the *E*_ave_ spectrum of different radii. The resonance wavelength increases proportionally with the resonator radius while the peak value of *E*_ave_ does not change significantly. This allows us to freely choose the geometric parameters in the experiment and smoothly tune the resonance wavelength to any position we need.

## 3 Fabrication

In the next step, we used an electron-beam lithography (EBL) based process to fabricate monolithic GaP metasurfaces in accordance with the optimized design. Hereby, the shared reactivity of GaP and metals like Cr and Al, which are commonly used as hard masks during etching with chlorine-based dry etching gases and additionally required wet etchants poses a challenge. This issue could in principle be overcome using an additional hard mask layer [18], [27], made of e.g. SiO_*x*_ or SiN_*x*_. However, this significantly complicates the process, particularly since toxic chemicals are commonly required to remove the hard mask after fabrication.

**Figure 3:**
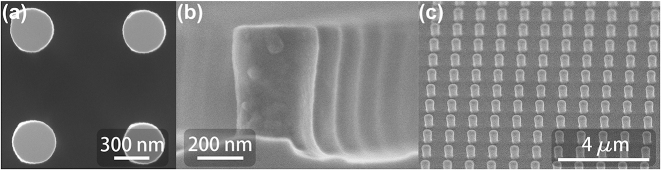
SEM images of the fabricated monolithic GaP metasurface. (a) Top-view SEM image of a fabricated metasurface obtained by processing with exposure doses during EBL of 240 μC/cm^2^. (b) The side view of the same metasurface as shown in panel (a). (c) Oblique-view SEM image of a typical fabricated metasurface.

In this work, we employed a simplified procedure using the e-beam resist directly as the etch mask (see Supplementary Material for further fabrication details). As a first step, the adhesion promoter (Surpass 4000, micro resist technology GmbH) was coated to ensure a good attachment between the GaP substrate and the resist. Then we spin-coated a commercially available double-side polished (110) GaP wafer (UniversityWafer, Inc.) with a 500 nm thick layer of the negative e-beam resist ma-N 2,405 and performed the electron-beam exposure and development. Due to the large band gap of GaP, the undoped GaP wafer is nonconductive at room temperature. A conductive polymer layer (mr-Conductive Layer, micro resist technology GmbH) is applied on the top of the resist to avoid charging of the sample during EBL. The high thickness of the resist results in a patterned resist layer thick enough to survive in etching with chlorine-based gases. However, the proximity effect can lead to the so-called footing, namely a residual around the bottom of the exposed resist mask. A plasma treatment was performed to remove the footing, thereby contributing to improved side-wall inclination of the final GaP structures (see Figure S2 in the Supplementary Material). Afterwards, inductively coupled plasma reactive-ion etching (ICP-RIE) was carried out using a gas mixture of 10 % chlorine, 30 % boron trichloride, 10 % nitrogen and 50 % Argon. The GaP crystals with (110) orientation did not show any anisotropic etching in relation to crystal planes here. As a final step, by thoroughly cleaning the sample with acetone and O_2_ plasma, the resist mask was completely removed. A top-view scanning electron microscope (SEM) image of a typical fabricated metasurface is shown in Figure 3a. The side view of the structure shown in Figure 3b exhibits a slight taper of the side wall as a result of the etching procedure. This leads to a blue shift of the resonance wavelength as compared to resonators with straight side wall and identical radius as seen in the top view. Figure 3c shows an oblique-view image of a fabricated GaP metasurface.

Using the described procedure, we fabricated three nanocylinder metasurfaces featuring a variation of the nanocylinder radius realized through a variation of the exposure dose during EBL from 160 μC/cm^2^ to 240 μC/cm^2^ in equidistant steps. In the following, we will refer to the metasurfaces as M1, M2, and M3, respectively. Their corresponding radii were measured as 160 nm, 175 nm, and 183 nm. The height of the nanocylinders was controlled to the range between 545 nm and 565 nm by tuning the etching duration. Also, the period of the array was ensured to be 960 nm via the high-resolution stage of the EBL system.

## 4 Linear and nonlinear optical properties

**Figure 4:**
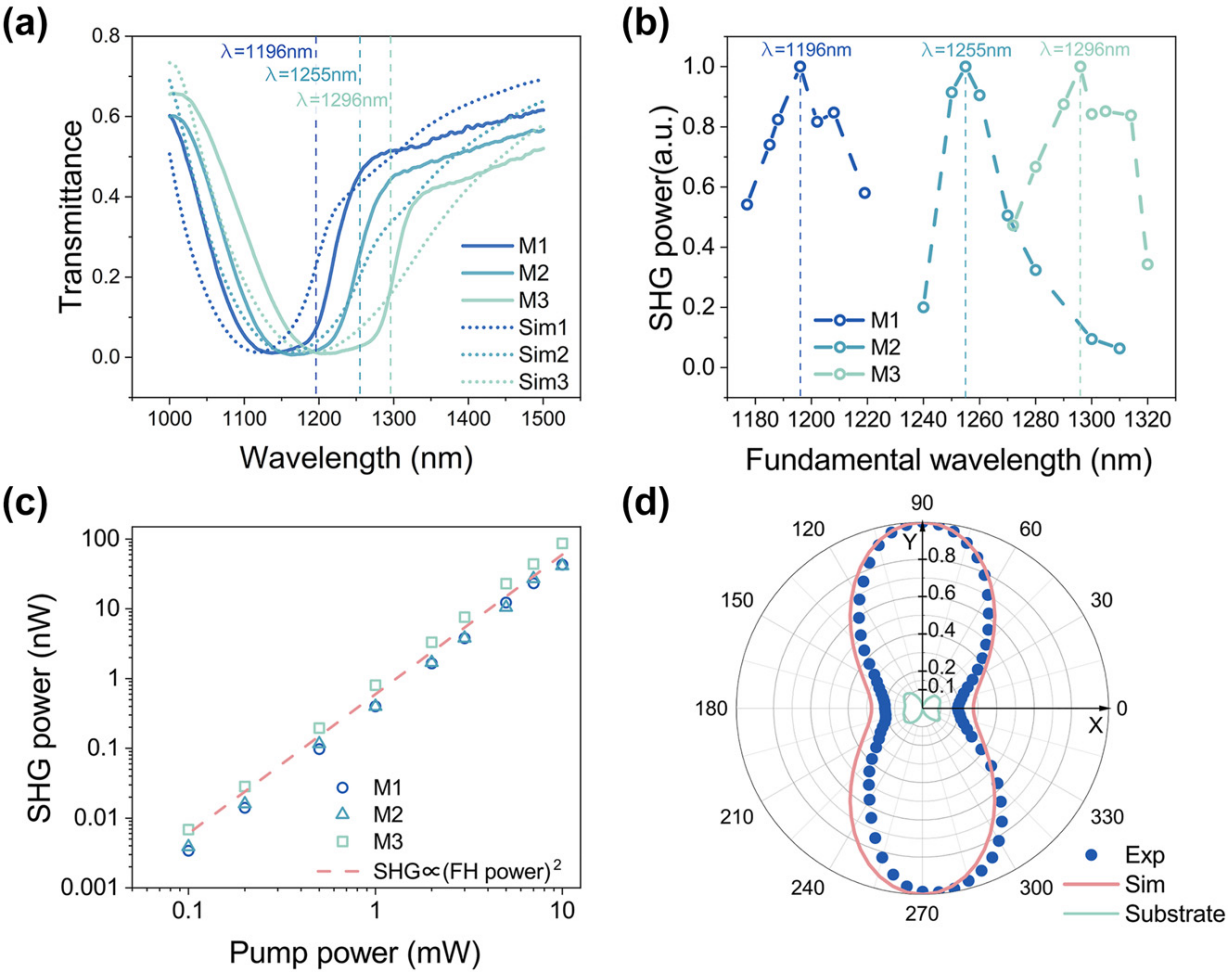
Optical properties of the fabricated monolithic GaP metasurface. (a) Linear-optical transmittance spectra of the three fabricated metasurfaces for unpolarized incident light. The dashed curves show numerically calculated spectra using measured geometry parameters for the three metasurfaces and taking the tapered shape of the nanocylinders into account. The vertical dashed lines mark the same position of the maximum of SHG signal as in (b) and the simulated position of the MD resonance for different fabricated geometry parameters. (b) Experimental SH intensities for different wavelengths at constant FH power. For each metasurface, the SH spectrum was normalized to its respective maximum. The wavelengths marked by the vertical dashed lines are the maximum position of the SHG signal and coincide with their counterparts in (a). (c) SHG power depending on FH average power measured at the wavelengths associated with the SHG maxima in (b). (d) Normalized SH power from M1 for a variation of the polarization direction of the FH beam (blue dots). For comparison, corresponding numerically calculated results for the optimized geometry are also added (red curve). Here 0° denotes the direction along the *x*-axis. The SH signal from the bare substrate at the same FH wavelength was normalized by the maximum of the SH signal from the metasurface.

To investigate the linear-optical properties of the fabricated metasurfaces, we recorded their transmission spectra using a self-built white-light spectroscopy setup (see Supplementary Material Figure S3). A tungsten halogen lamp was used as a light source and the transmittance was recorded using an optical spectrum analyzer (Yokogawa AQ6370B). Due to the four-fold symmetry of the metasurface structure, no strong polarization dependence of the transmittance is expected and measurements were performed with unpolarized light. Weak focusing means the light is not tightly focused, which is the method we used to collimate the light. Moreover, using weak focussing and appropriate apertures, the light impinging on the sample was restricted to near-normal incidence. The influence of the GaP-air interface at the backside of the wafer was referenced out by dividing by the root of the measured transmittance of the bare GaP wafer with the same thickness. Figure 4a shows the corresponding linear-optical transmittance spectra of the three metasurfaces. All metasurfaces exhibit a broad minimum in the 1,000 nm–1,300 nm range. As the nanocylinder radius increases from M1 to M3, a redshift of this minimum is observed. For comparison, numerically calculated spectra for the corresponding radii are also included. Here, the tapered shape of the nanocylinders (see Figure 3b) was taken into account. While we observe a good qualitative agreement between measured and numerically calculated spectra, in the measured spectra the minimum is broader than observed in the numerical results and the second, smaller minimum associated with the MD resonance cannot be discerned clearly. As a likely explanation, the two resonant features expected from simulations are merged in the experimental spectra, forming one broad minimum. Based on this explanation, we expect the maximum SHG close to the long-wavelength slope of the resonant features (see the vertical dashed lines). The moderate deviations between the experimentally measured and numerically calculated spectra are likely due to fabrication imperfections such as roughness and nonuniformity, as well as to the finite numerical aperture (NA = 0.04) used in experiments as compared to assumed plane wave illumination in the numerical model.

Next, we performed a nonlinear optical characterization of the fabricated samples using a home-built SHG microscope setup. To this end, the idler beam from the INSPIRE HF100 optical parametric oscillator pumped by a tunable femtosecond Ti:Sapphire laser (Spectra Physics Mai Tai) was focused onto the back side of the sample. The pulse duration was approximately 200 fs. A sequence of a half-wave plate and a linear polarizer was used to control the power of the incident FH light. The FH was set to be polarized along the *y*-direction. After filtering out the FH behind the sample, the signal was recorded using an electron-multiplying CCD camera (iXon3 EMCCD, Andor). The incident FH power was kept constant at 5 mW. Figure 4b shows the relationship between the SH power and wavelength for all three metasurfaces samples. For each metasurface, the obtained SH spectrum was normalized to its respective maximum. The maximum SH power is reached around 1,196 nm (M1), 1,255 nm (M2), and 1,296 nm (M3).

The dependence of SH power on FH power is shown in Figure 4c, showing clearly quadratic behavior. The SHG conversion efficiencies *η* and nonlinear coefficient *ξ*_SHG_ are defined as follows:

$$\eta =\frac{P_{\text{ave}}^{\text{SH}}}{P_{\text{ave}}^{\text{FH}}}$$

$$\xi_{\text{SHG}}=\frac{P_{\text{pk}}^{\text{SH}}}{{\left(P_{\text{pk}}^{\text{FH}}\right)}^{2}}$$

where $P_{\text{ave}}^{\text{SH}}$ and $P_{\text{ave}}^{\text{FH}}$ are the average power of the SH and FH wave, $P_{\text{pk}}^{\text{SH}}$ and $P_{\text{pk}}^{\text{FH}}$ are the estimated peak power of the SH and FH wave. For an FH average power of 5 mW ( $P_{\text{pk}}^{\text{FH}}=625\;W$ or 1.1 GW/cm^2^ peak intensity, estimated based on the 6 μm excitation spot diameter obtained from the camera), the near-infrared to visible conversion efficiencies *η* of the three metasurfaces at their corresponding resonance wavelengths are determined as 2.43 × 10^−5^, 2.28 × 10^−5^, and 4.95 × 10^−5^ respectively. To determine these efficiencies, the transmittance of all optical components that affect the power measurement, the reflection from the sample back interface, and the collection efficiency of the collection lens were taken into account. The SH produced by the substrate was not corrected for, because it only accounted for 1.3 % of the total forward SH power for *y*-polarized FH incidence. Although the experimentally observed values are one order of magnitude smaller than numerical results, these efficiencies are already comparable to previous thin-film III–V material SH metasurfaces [11], [41]. Notably, M3 shows the highest conversion efficiency. Nonlinear simulations using the same geometric parameters as for the calculation of the linear-optical spectra yield conversion efficiencies of three metasurfaces at their corresponding MD resonance wavelengths of 5.80 × 10^−4^, 7.08 × 10^−4^, and 7.02 × 10^−4^, respectively (see Supplementary Material for details). Additionally, in order to eliminate the influence of pump power on SHG performance, the *ξ*_SHG_ of the three metasurfaces was calculated as 3.89 × 10^−8^W^−1^, 3.65 × 10^−8^W^−1^, and 7.92 × 10^−8^W^−1^, respectively.

As a next step, we studied the relationship between SH power and FH polarization on M1. A half-wave plate located behind a linear polarizer was used to change the polarization of the incident FH light. Corresponding results are shown in Figure 4d, exhibiting a clear maximum of the SH power for *y*-polarized FH. The polarization dependency of the SH signal from the bare substrate was measured next to M1 at the same FH wavelength and plotted in Figure 4d, which shows a very limited contribution of the SH signal from the substrate. Moreover, the normalized data shows good agreement with the simulation results of polarization dependence of forward SH power for the optimized geometry.

**Figure 5:**
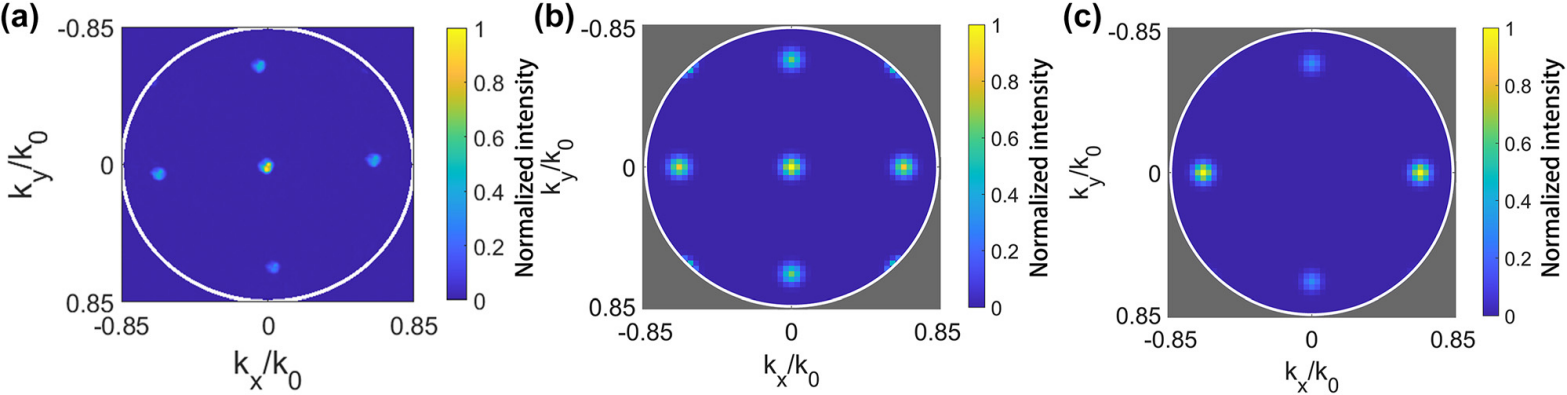
Experimentally measured and numerically simulated back-focal plane image of the emitted SH for the metasurface. (a) Experimentally measured back-focal plane image of the emitted SH for M1 for a FH wavelength of 1,196 nm. The highest numerical aperture that can be collected is 0.85. (b)–(c) Numerically simulated back-focal plane image of the emitted SH for the metasurface with (b) (110) and (c) (100) GaP crystal orientation and identical geometry parameters (*r* = 150 nm, *h* = 540 nm, *p* = 960 nm) for an FH wavelength of 1,230 nm. The white circle corresponds to NA = 0.85. The area where NA > 0.85 is shaded in gray for numerical data.

One of the most important reasons for using (110) crystal orientation is the opportunity to reach efficient SHG in the normal direction with respect to the sample plane [3], [36]. In order to investigate the SH emission pattern for the GaP metasurfaces, we recorded back-focal plane images of the emitted SH for sample M1 under *y*-polarized FH excitation. These results are shown in Figure 5a. The numerical aperture (NA) of the collection lens was 0.85. Five diffraction orders were collected, among which the 0th order had the highest intensity. To further analyze the directionality of SH emission, we used the vector angular spectrum method [42] to numerically analyze the output SH electric field of the metasurface for (110) and (100) crystal orientation and plotted the simulated intensity in the back-focal plane as shown in Figure 5b and c, respectively. The geometrical parameters of the metasurface are the same as in Figure 2a, and the fundamental wavelength is 1,230 nm. The metasurfaces were excited by *y*-polarized FH incident light. Figure 5b shows that for the metasurface made of (110) GaP crystal, among the SH diffraction orders that can be collected within the NA of 0.85 used in experiments, the intensity of the 0th order is relatively high. In contrast, the SH intensity directed into the 0th order of the metasurface made of (100) GaP crystal is negligible. Note, however, that in both cases more energy is distributed into higher diffraction orders that cannot be collected experimentally (see also Supplementary Material). This supports our choice of the (110) GaP crystal for metasurface fabrication and underpins their suitability for applications that require on-axis emission of SHG.

## 5 Conclusions

In this work, we successfully demonstrate SHG at visible frequencies from monolithic GaP-on-GaP substrate nanocylinder metasurfaces. The metasurfaces are designed to support well-confined MD-dominated Mie-type resonances in the near-infrared spectral range, providing significant enhancement of the fundamental harmonic electric field. The exact resonance wavelength can be tailored via the metasurface geometrical parameters. To facilitate SH emission along the optical axis, a wafer with (110) crystal orientation was chosen for fabrication. We fabricated three metasurfaces featuring a systematic variation of the nanocylinder diameter. Linear and nonlinear optical characterization of the metasurfaces confirmed the excitation of Mie-type resonances as well as significant SHG enhancement when the fundamental harmonic wave is tuned to the resonance wavelength of the respective structure. We measured a high nonlinear coefficient of 7.92 × 10^−8^W^−1^ and conversion efficiency of 4.95 × 10^−5^ at 5 mW pump power enabled by a combination of the relatively strong resonant confinement in the high-refractive-index nanostructure, the high *χ* ^(2)^ of GaP and its large transparency range. Back-focal plane imaging of SHG emission shows that a significant portion of the SH is emitted normally out of the metasurface plane along the optical axis. Altogether, our results indicate that monolithic GaP-on-GaP metasurfaces can act as an excellent substitute for nanostructures from poorly available thin-film GaP for enhancing, tailoring, and harnessing *χ* ^(2)^ nonlinear processes. As such, they can promote wider exploitation of the outstanding combination of the linear and nonlinear material properties of GaP for many applications in nonlinear and quantum optics [43]. Moreover, the improved availability of resonant nanophotonic structures made from single crystalline GaP could propel several other subfields of nanophotonics, including in the areas of high-index gratings, photocatalysis enhancement, and light-emitting metasurfaces.

## 6 Methods

### 6.1 Numerical simulations

Linear and nonlinear simulations were performed by means of the RF module implemented in the commercial software COMSOL Multiphysics 5.6, which is based on the finite element method. Nonlinear simulations were conducted in the undepleted pump approximation [44]. To this end, we first simulated the fundamental electromagnetic field inside the unit resonator cell with a linear port excitation. The GaP substrate is considered as semi-infinite, with the port located inside the substrate and the launched plane wave traveling in positive *z*-direction. The geometry parameters (*r*, *h*, and *p*) were swept to find a resonance with a significant electric field enhancement. The complex refractive index of GaP was modeled using experimental data from Ref. [39]. The incident light intensity is set to *I*_0_ = 1 GW/cm^2^. Secondly, the electric field inside the resonator was used to calculate the nonlinear polarization density as the source of the SH field. Then, another linear simulation was performed at the SH frequency, utilizing the non-linear polarization density induced by the FH field. Then, another linear simulation was performed at the SH frequency, utilizing the non-linear polarization density as the source.

## Supplementary Material

Supplementary Material Details
