# Supplementary material for: Second harmonic generation in monolithic gallium phosphide metasurfaces
Source: Nanophotonics. 2024 Jul 11;13(18):3311–9. doi: 10.1515/nanoph-2024-0177 (PMC11501941; doi:10.1515/nanoph-2024-0177)
Supplement: Supplementary file 1 — Supplementary Material Details [file j_nanoph-2024-0177_suppl_001.pdf]

Supplementary Material for:

Second Harmonic Generation in Monolithic Gallium Phosphide Metasurfaces

Muyi Yang[1,2,3*], Maximilian A. Weissflog[2,3], Zlata Fedorova[1,2] , Angela I. Barreda[1,2,5], Stefan Börner[2], Falk Eilenberger[2,3,4], Thomas Pertsch[2,3,4], Isabelle Staude[1,2,3]

[1]Institute of Solid State Physics, Friedrich Schiller University Jena, Max-Wien-Platz 1, 07743 Jena, Germany

[2]Institute of Applied Physics, Abbe Center of Photonics, Friedrich Schiller University Jena, Albert-Einstein-Str. 15, 07745 Jena, Germany

[3]*Max Planck School of Photonics, Hans-Knöll-Straße 1, 07745 Jena, Germany*

[4]*Fraunhofer Institute for Applied Optics and Precision Engineering IOF, Albert-Einstein-Straße 7, 07745 Jena, Germany*

[5]*Group of Displays and Photonics Applications, Carlos III University of Madrid, Avda. de la Universidad, 30, Leganés, 28911 Madrid, Spain*

[*]Corresponding author: muyi.yang@uni-jena.de

Contents

1. Fabrication details

2. Measurement setup

3. Second order nonlinear electric susceptibility of (110) GaP

4. Comparison with front illumination

5. Simulation of SH in the back-focal plane for different incident polarizations and crystal orientations

References

1. Fabrication details

In this work, we used commercially available (110) oriented double-side polished GaP wafer (UniversityWafer, Inc.) with the thickness of 450 μm as starting material. As a first step, the GaP wafers were thoroughly cleaned using a combination of washing them with acetone and isopropanol and an oxygen plasma clean. This is vital for preconditioning the wafer surface regarding its adhesion properties. The dehydration baking of 200°C for 10 minutes was carried out to further ensure a good attachment of the resist. Next, the adhesion promoter (Surpass 4000, micro resist technology GmbH), negative electron-beam resist (ma-N 2405, micro resist technology GmbH) and conductive polymer (mr-Conductive Layer, micro resist technology GmbH) were sequentially applied on GaP samples. The thickness of the resist was about 500nm with a coating speed of 3000 revolutions per minute. The electron-beam exposure was carried out with an eLINE Plus System (Raith GmbH) at 30 kV acceleration

voltage and using the 10 μm aperture. In this case the exact current is about 40 pA. The size of single working field is 50 μm. After 50s development by ma-D 525(micro resist technology GmbH), we observed a significant footing effect, namely undesired resist residuals close to the exposed structures (see Figure S2).

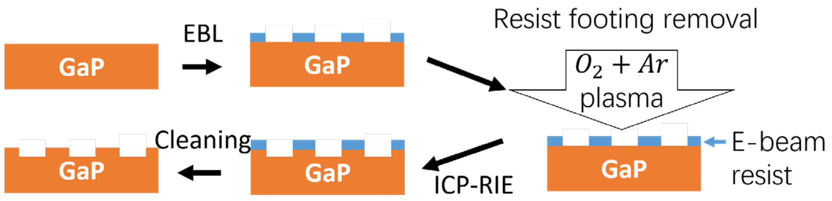

Figure S1: Process flow of the demonstrated fabrication process. The blue structure on GaP substrate stands for e-beam resist.

To remove the footing, we performed a plasma treatment with 95% Ar and 5% O_2, applying radio frequency (RF) power at low pressure using a PlasmaPro 100 Cobra System (Oxford Instruments). The low pressure helps to maintain good directionality and high energy of the ion bombarding on the sample surface, which reduces isotropy in etching process. With 15s of the treatment at a etch rate about 5 nm/s, the resist footing can be removed, while the shape of the resist was well preserved. The resist structure after footing removal was subsequently used as a soft mask to transfer the exposed pattern into the GaP. The actual GaP etching was conducted in the same device using an etch gas combination of 10% Cl_2, 30% BCl_3, 10% N_2 and 50% Ar at a temperature of 20°C, resulting in an etch rate of about 500nm/min. The GaP sample was etched on a 4-inch silicon dummy wafer. As a last step, the remaining resist was removed with acetone and an additional O_2 plasma cleaning.

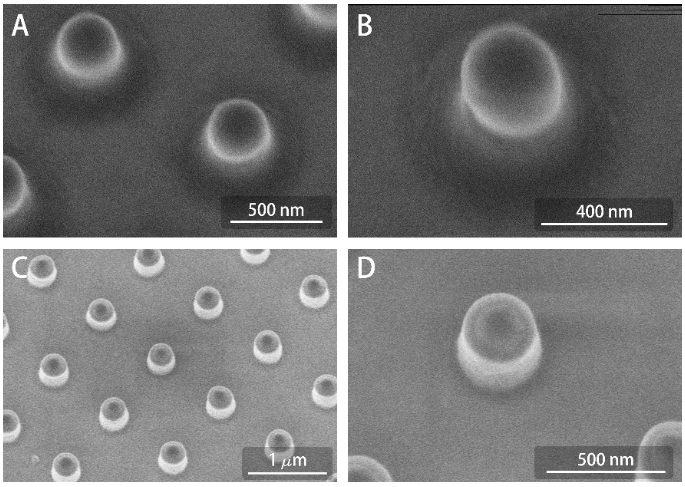

Figure S2: SEM images of the developed resist structure before and after the footing removal treatment. (A, B) Before the treatment. (C, D) After the treatment.

2. Measurement setup

Figure S3 shows the white-light spectroscopy setup that was employed for the measurement of the linear-optical transmittance spectra. As the measured wavelength is larger than the period of the

metasurface, there is no diffraction order higher than zero. The sample was illuminated by unpolarized white light from the back side. A set of lenses projected the image of the sample surface onto the field stops to select the area to be measured.

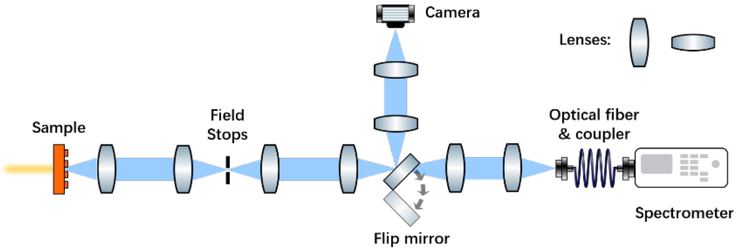

Figure S3: Sketch of the white-light spectroscopy setup employed for linear-optical transmittance measurements.

The nonlinear-optical measurements were performed using a self-built microscope setup as shown in Figure S4. As excitation source, an INSPIRE HF100 optical parametric oscillator (OPO) was used. The OPO was pumped by a Mai Tai HP femtosecond Titanium:Sapphire laser with central wavelength $\lambda_c = 820\ \mathrm{nm}$, pulse duration 100 fs, average power 2.75W and repetition rate of 80 MHz. The OPO idler output with pulse duration of 200fs was focused by an objective (Mitutoyo) with numerical aperture NA = 0.2 onto the sample. The polarization and power of incident light were controlled by two half-wave plates and a linear polarizer before the objective. The SH signal was collected by an objective with high numerical aperture of NA=0.85 (Zeiss EC Epiplan 422090 99092). After filtering out the FH using a short-pass filter (SPF), the image was acquired by an electron-multiplying CCD camera (iXon3 EMCCD, Andor). In the back-focal plane measurement, an additional lens (Lens 1) was included after the collection objective to project the back-focal plane image onto the camera.

The incident power was measured after the second half-wave plate and before the first objective. The SHG power was measured after the SPF and before Lens2. The optical losses of all the remaining components were taken into account for the calculation of SHG conversion efficiency as follows. The transmittance of the Mitutoyo objective at the FH wavelength range (1196 nm to 1296 nm) is 0.7. The reflectance of the back side of the GaP sample in the FH wavelength range is 0.23. The transmittance of the Zeiss objective in the SH wavelength range (598 nm-648 nm) is 0.92. The transmittance of the SPF at the SH wavelength range is between 0.9 and 0.97. Furthermore, the far-field simulation showed the SH power collected by the objective (NA=0.85) accounts for 21.7% of the emitted SH power in the 4π solid angle. The remaining SH power is emitted into higher diffraction orders in forward and backward directions. This was also considered for the conversion efficiency calculation.

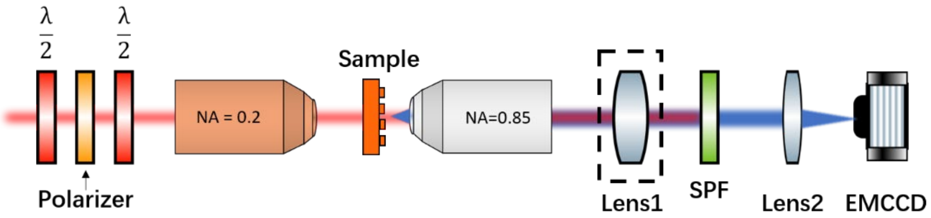

Figure S4: Sketch of the experimental setup used for SHG in transmission mode. From left to right: half-wave plate and linear polarizer to control the FH power, half-wave plate to tune the polarization, focusing objective, sample, collection objective, lens 1 to project the back-focal plane onto the camera, short pass filter (SPF), lens 2 to focus the light to the camera, and EMCCD for detection.

3. Second order nonlinear electric susceptibility of (110) GaP

Because of the zincblende structure of GaP crystal, the second order nonlinear electric susceptibility tensor $\chi^{(2)}$ of (110) crystal orientation is given as:

$$\begin{pmatrix} P_x^{(2)} \\ P_y^{(2)} \\ P_z^{(2)} \end{pmatrix} = \varepsilon_0 \chi^{(2)} \begin{pmatrix} E_x^2 \\ E_y^2 \\ E_z^2 \\ 2E_yE_z \\ 2E_xE_z \\ 2E_xE_y \end{pmatrix} = 2\varepsilon_0 \mathrm{d}_{14} \begin{pmatrix} 0 & -1 & 1 & 0 & 0 & 0 \\ 0 & 0 & 0 & 0 & 0 & -1 \\ 0 & 0 & 0 & 0 & 1 & 0 \end{pmatrix} \begin{pmatrix} \mathrm{E_x^2} \\ \mathrm{E_y^2} \\ \mathrm{E_z^2} \\ 2\mathrm{E_yE_z} \\ 2\mathrm{E_xE_z} \\ 2\mathrm{E_xE_y} \end{pmatrix}$$

where d_{14} is the nonlinear coefficients. The value of d_{14} used here is 37 pm/V [1]. From this, the nonlinear polarization density can be directly calculated.

4. Comparison with front illumination

To compare with the simulations performed assuming illumination from the back side of the sample, we also considered the linear fields for front illumination, i.e. with the pump light incident from the metasurface side of the wafer. The light intensity is same as that at the back illumination discussed in the main text. The same geometric parameters ($r = 150\ \mathrm{nm}, h = 540\ \mathrm{nm}, p = 960\ \mathrm{nm}$) as in the examples in the main text were used for the front illumination simulation. As expected for reciprocal structures and shown in Figure S5A, the metasurface showed the same transmittance spectra as when illuminated from the back. However, the average electric field enhancement is significantly reduced due to the reflection caused by the change in the refractive index at the air-GaP interface. To take into account the reflection at the air-GaP interface for incidence from the back side of the sample, we multiplied the average FH enhancement for back illumination by the transmittance of the air-GaP interface of T = 0.74. The results are also included in Figure S5A (dashed line), where we observe an average enhancement of 10.8 at λ = 1230 nm. Figure S5B show the cross-section of the electric-field enhancement $\left(\frac{|\vec{E}|}{|\vec{E_0}|}\right)^2$ at the wavelength of 1230 nm with front illumination. The $\left(\frac{|\vec{E}|}{|\vec{E_0}|}\right)^2$ is much lower in the resonator when compared with backward illumination.

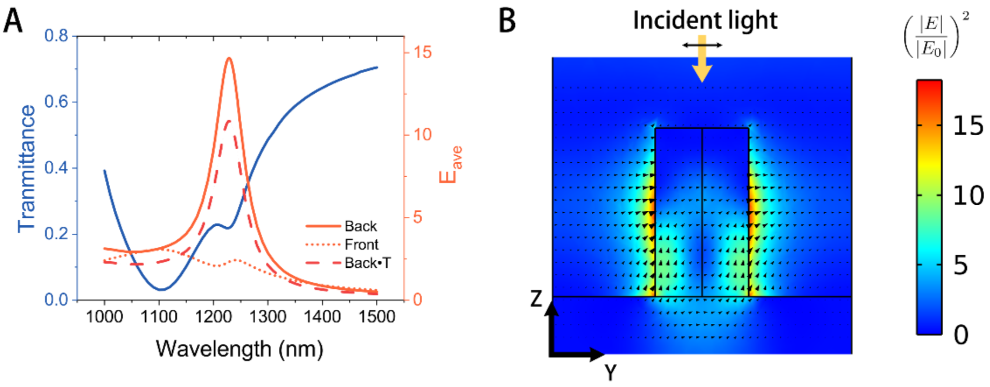

Figure S5: (A) Comparison of simulated transmittance spectra (blue curve) and the field enhancement E_{ave} for front and back illumination for a metasurface with identical geometric parameters as in the main text. As expected, the transmittance is the same for front and back illumination of the sample. E_{ave} for front and back illumination is plotted as dotted and solid red lines, respectively. The E_{ave} of back illumination corrected by the transmittance T of the wafer backside is plotted as a dashed line. The front illumination has a much lower E_{ave} at 1230 nm. (B) Cross-section of the electric-field enhancement $\left(\frac{|\vec{E}|}{|\vec{E_0}|}\right)^2$ in the *yz* plane through the centre of a nanocylinder of the metasurface at a wavelength of 1230 nm for the case of front illumination. The arrows size and direction denote the value of $\left(\frac{|\vec{E}|}{|\vec{E_0}|}\right)^2$ and direction of the electric field vector, respectively.

5. Simulation of SH in the back-focal plane for different incident polarizations and crystal orientations

In order to investigate the effect of the FH polarization on the SH emission directionality, corresponding SH back-focal plane images were simulated and plotted in Figure S6 A-E for optimized geometric parameters of the metasurface ($r = 150\ \text{nm}, h = 540\ \text{nm}, p = 960\ \text{nm}$) and different incident polarizations. Here, the FH wavelength is 1230 nm. For comparison, numerically calculated SH back-focal plane images for (100) crystal orientation and *y*-polarization are included as Figure S6F. Note that in both cases, notable SH is emitted into higher diffraction orders not collected in the experiment. To quantify the amount of SH directed in forward direction, we calculated the fraction of power into the 0th diffraction order, $P^{(0)}$, as well as more generally in all forward diffraction orders P_{forward} in relation to the total SH power emitted in all directions, including backward SH emission. Corresponding results are shown in Figure S6G. The case of (110) crystal orientation and *y*-polarized excitation is found to exhibit the highest power percentage of 1.67% emitted into the 0th diffraction order with almost 79.2% of the power going in all forward diffraction orders. For (100) crystal orientation(shaded yellow in S6G), the former value is negligible.

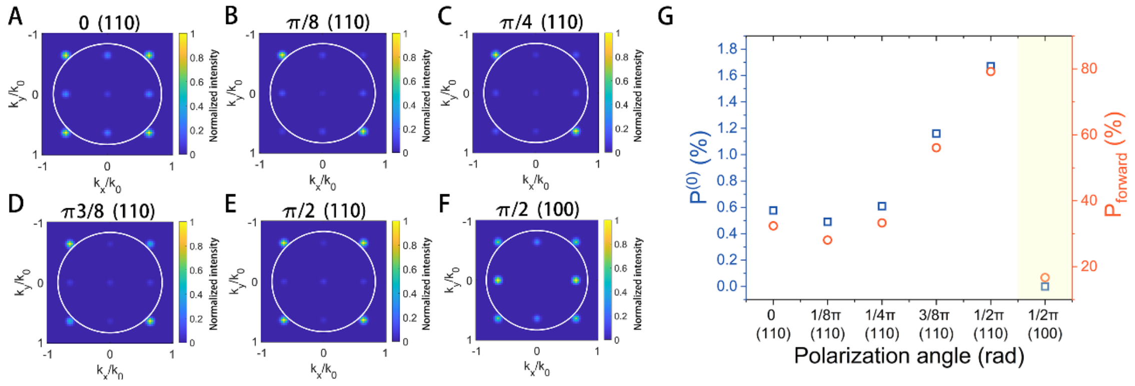

Figure S6: (A)-(F) Simulated back-focal plane images of the SH signal for the geometrically optimized metasurface ($r = 150$ nm, $h = 540$ nm, $p = 960$ nm) at the FH wavelength of 1230nm with different incident polarizations and crystal orientations. The white circle represents the range of NA=0.85. The polarization angle, θ, is defined by the angle between the electric field vibration direction and the *x*-axis. (A-E) (110) crystal orientation with (A) θ=0, (B) θ=$\frac{\pi}{8}$, (C) θ=$\frac{\pi}{4}$, (D) θ=$\frac{3\pi}{8}$, (E) θ=$\frac{\pi}{2}$. (F) (100) crystal orientation, θ=$\frac{\pi}{2}$. (G) The percentage of power of 0th diffraction order, $P^{(0)}$, in the total SH power and the percentage of forward SH power, $P_{forward}$, in the total SH power for the 6 different studied configurations described before.

Reference

[1]. I. Shoji, T. Kondo, A. Kitamoto, M. Shirane, and R. Ito, "Absolute scale of second-order nonlinear-optical coef-ficients," *Journal of The Optical Society of America B-optical Physics*, vol. 14, no. 9, p. 2268, Sep. 1997. doi:10.1364/josab.14.002268.
